# Family food environment factors associated with obesity outcomes in early childhood

**DOI:** 10.1186/s40608-019-0241-9

**Published:** 2019-06-03

**Authors:** Nikki Boswell, Rebecca Byrne, Peter S. W. Davies

**Affiliations:** 1 The University of Queensland, QLD, Brisbane, Australia; 2 Queensland University of Technology, QLD, Brisbane, Australia

## Abstract

**Background:**

In attempting to gain understanding of the family food environment (FFE), as a central context for the development of obesity and obesogenic eating behaviours during early childhood, attention has largely focused on the relationships of individual variables. This fails to capture the complex combinations of variables children are exposed to. To more authentically reflect the impact of the FFE on the development of obesity and obesogenic eating behaviours during early childhood, this study aims to derive composites of FFE variables using factor analysis.

**Methods:**

FFE and eating behaviour data were available from 757 Australian children (2.0–5.0 years) via a parent-completed online survey. Children were categorised as normal weight, overweight or obese, based on parent-reported anthropometry (underweight children were excluded).

**Results:**

Eight FFE factors were derived. Scores for factors ‘Negative Feeding Strategies’ and ‘Negative Nutrition Related Beliefs’ increased with child BMI category, while ‘Use of TV and devices’ and ‘Parent’s Nutrition Knowledge’ decreased. The FFE factor ‘Negative Feeding Strategies’ was positively associated with food fussiness, food responsiveness and slowness in eating, and negatively associated with parent body mass index (BMI) score. The FFE factor ‘Negative Nutrition Related Beliefs’ was positively associated with food responsiveness, as well as positively with parent BMI, male children, breastfeeding less than 6 months, and low-income status. The FFE factor ‘Television (TV) and devices’ was only positively associated with residing in a capital city. The FFE factor ‘Parent’s Nutrition Knowledge’ was negatively associated with slowness in eating, breastfeeding less than 6 months and low-income status, and positively with parent stress and residing in a capital city.

**Conclusion:**

Consideration of the composite effect of FFE on child’s eating behaviours and obesity outcomes is important in guiding future research and obesity prevention initiatives by providing a more authentic picture of the FFE children are exposed to. Examining factors of FFE variables in conjunction with psycho-social variables, further articulates the reciprocal influence of these variables on environmental constructs thus assisting in understanding of inequitable distribution of obesity risk.

***keywords:**

childhood obesity, eating behaviours, early childhood, Family Food Environment, Factor Analysis,

## Background

Childhood obesity is a multifactorial condition which involves interaction between genetics, environments and behavioural responses [1, 2]. A key example of this is the interaction between children’s eating behaviour and the family environment in the development of childhood overweight and obesity [3, 4]. Eating behaviours such as food responsiveness and enjoyment of food, referred to as food approach eating behaviours, are positively associated with obesity development, while food avoidance eating behaviours, such as satiety responsiveness, food fussiness and slowness in eating, have been seen to be negatively associated with obesity development [3, 4]. Given this, much attention has focused on initiatives which aim to alter ‘obesogenic’ behaviours and obesity development via environmental modifications [5, 6]. For such interventions to be effective, however, a thorough understanding of environmental contexts and their influence on obesity and behavioural intermediaries is necessary.

Whilst environmental influences are considered to operate at multiple levels, as conceptualised through the socio-ecological model, for children, the family food environment (FFE) has been seen to explain the greatest variance in obesity, compared with school and neighbourhood level influences, and is a prime context in which children’s eating behaviours emerge [7–9]. As the ‘first ecological niche of children,’ it is within the confines of the FFE that parents impose socio-cultural values and practices around food and eating occasions (interpersonal influences of the socio-ecological model), as regulated by the structural boundaries and resource limitations of the home (micro-environment influences of the socio-ecological model). For instance, interpersonal influences such as parental use of controlling feeding practices have been associated with increased body weight in children as well as tendencies towards obesogenic eating behaviours [10–13]. Similarly, micro-environment influences such use of television (TV) during meals and availability of fruit and vegetables within the home, have been associated with obesogenic eating behaviours and increased body weight [14–17].

While the literature to date has highlighted the potential importance of numerous FFE variables (e.g. parental feeding strategies, frequency of family meals, the use of TV and electronic devices during meals, cooking and home resources, parent’s food and nutrition related beliefs, parent’s cooking and shopping skills, parent’s nutrition knowledge) in the development of obesity and obesogenic eating behaviours, the collective influence of these variables, has not been considered [6, 13, 16, 18–20]. Considering FFE variables independently limits understanding of the collective impact of variables, as a more authentic reflection of the environmental context in which obesity and obesogenic eating behaviours develop. For instance, while non-responsive feeding strategies (e.g. parental use of pressure, bribes, coercion and control) have been seen to be associated with childhood obesity and ‘obesogenic’ eating behaviours, research has not examined the occurrence of other FFE variables, such as use of TV during meals, the frequency of family meals, availability of fruit and vegetables or parent’s nutrition knowledge, which may partner with non-responsive feeding strategies to have an impact on obesity development [3, 4, 6, 12, 13, 18, 21–23]. Additionally, consideration has not been given to exploring differences in collections of FFE variables based on psycho-social factors such as income, parent’s marital status, parent’s depression, anxiety and stress, region of residence, or parent’s body mass index (BMI), which are likely to have a significant impact on the FFE and consequently may contribute to explanations of inequitable distribution of obesity risk within the population.

Given this, this study aims to use factor analysis to derive composites of FFE variables, to provide a more authentic reflection of FFE exposure during early childhood in Australia. Highlighting FFE variables that appear to group together in this way offers a novel perspective from which to further examine the development of obesity and obesogenic eating behaviours. Additionally, since psycho-social factors such as income, parent’s marital status, parent’s depression, anxiety and stress, and parent’s BMI, are likely to have a distinctive impact on the FFE constructed, relationships between these variables and FFE factors will be examined.

## Methods

Between July and November, 2016, Australian parents of children aged 2.0–5.0 years self-enrolled to complete an online, cross sectional survey. Participants self-selected to enroll in the survey through advertising on the social media website Facebook®. Children were excluded from this study if they were reported to have a medical condition likely to affect their growth, development or metabolism. In the instance that a parent had more than one child within the target group, parents were asked to refer to the child whose birthday occurred next. No incentives were offered for participation in this survey. Participants were asked to use household measures (e.g. bathroom scales/ household tape measure) to report their weight and height, and that of their child, which were subsequently used to calculate BMI categories (according to the 2000 CDC growth charts for children; BMI categories as per Cole 2000 and 2007) [24, 25]. As child height and weight were by parental report it was deemed necessary to screen the data for biologically implausible values. The process used to screen these data for biologically implausible values has previously been reported [26].

Children’s eating behaviours were measured using sub-scales of the Children’s Eating Behaviour Questionnaire (CEBQ; enjoyment of food, food responsiveness, satiety responsiveness, food fussiness and slowness in eating). Internal reliability of these scales for this sample has previously been reported (see Boswell, et al., 2018) and ranged from Cronbach α 0.921–0.677 [26]. The CEBQ has been well validated across the literature including in Australian samples of young children [27, 28].

Demographic variables recorded included child’s age, recorded to the nearest half year, the gender of the parent that completed the questionnaire, the child’s gender, family income reported as low (less than AU$40,000), middle (AU$40,000 – AU$100,000) or high (more than AU$100,000), the duration the response child was breastfed, and Australian state and region of residency (based on Rural, Remote and Metropolitan Areas (RRMA) classification) [29]. Parent’s depression, anxiety and stress levels, as an important covariate of childhood obesity identified in these data previously (see Boswell, et al., 2018) were measured using the Depression, Anxiety and Stress Scale [DASS-21] [26, 30]. Variables conceptualized within the FFE, as aligning with interpersonal and micro-environment levels of the socio-ecological model, were measured as per the scales described below and screened for internal reliability.

### Parent’s feeding practices and structure

The 8 Feeding Practice and Structure Questionnaire [FPSQ-28] sub-scales, as validated in a sample of Australian children 2–5 years, were scored as per the relevant literature [31]. All FPSQ-28 sub-scales produced a Cronbach α above 0.6 (Reward for Behaviour [4 items; Cronbach α 0.820], Reward for Eating [4 items; Cronbach α 0.672], Persuasive feeding [6 item; Cronbach α 0.803], Covert Restriction [4 items; Cronbach α 0.808], Overt Restriction [4 items; Cronbach α 0.605], Structured Meal Setting [3 items; Cronbach α 0.865], Structured Meal Timing [3 items, Cronbach α 0.670], and Family Meal (single item).

### The frequency of family meals

The frequency of family meals was measured using three items, reflecting breakfast, lunch and dinner, to create a total frequency of family meal score (out of 21).

### General nutrition knowledge

A general nutrition knowledge score (out of 13) was created based on a general knowledge questionnaire guided by the works and recommendations of Parmenter, et al., (1999), and a similarly adapted version validated for use with Australian adults [32, 33].

### Nutrition related beliefs

Four nutrition related belief items were measured (‘Eating healthy is expensive,’ ‘It takes too long to prepare a healthy meal,’ ‘Healthy food doesn’t taste good,’ Nutrition is important to your family’). Items were devised based on key barriers to healthy eating qualitatively themed from a sample of Australian adults and phrased as a belief by assigning attributes to identified barriers towards healthy eating [34, 35]. Each item was scored individually as a categorical variable. A higher score on this scale reflects poorer nutrition related beliefs.

### Parental depression, anxiety and stress

Parental depression, anxiety and stress was measured using the DASS-21, depression, anxiety and stress scale [30]. The DASS-21 is a 21 item self-report questionnaire designed to measure the severity of a range of symptoms common to depression, anxiety and stress over the previous week [36]. Data from this study showed high internal reliability for each scale; Stress [7 items; Cronbach α 0.837] Anxiety [7 item; Cronbach α 0.742] Depression [7 items; Cronbach α 0.886]. Each DASS scale was examined for normality (skewness and kurtosis between 1 and − 1). Depression and anxiety scales were deemed skewed so transformed accordingly, however, the stress scale was normally distributed.

### Home resources and Parent’s personal skills

Parent’s cooking and grocery shopping skills, along with the availability of fruit and vegetables within the home, cooking facilities, food storage facilities, and the use of TV/electronic devices during meals (3 separate items: family use, child use, and adult use), were reported as categorical variables on Likert or on nominal scales.

### Ethics approval

Ethical approval for this research project has been granted through The University of Queensland (approval number 2016000860).

### Statistical method

The distribution of predictive variables was examined for multicollinearity and normality (skewness and kurtosis between 1 and − 1). Factor analysis, with Kaiser-Mayer-Olkin measure and Bartlett test of sphericity was run to create composites of FFE variables, with orthogonal rotation (Varimax) performed to determine how strongly a variable contributed to a FFE factor, based on eigenvalues >1. Intra-correlation between variables was assessed using Kaiser-Mayer-Olkin measure, with values >0.5 considered to indicate good intra-correlation [37, 38]. Variables with value <0.5 were removed and analysis rerun, as recommended by Fields, 2005 [37]. Items were loaded on a factor if they had a positive or negative correlation >0.25 with that factor and named descriptively [39, 40].

To examine whether BMI categorization showed linear associations with derived FFE factors, a one-way between-groups multivariate analysis of variance (MANOVA) was performed. Pillai’s Trace was examined for significance, homogeneity of variance assumption examined with Levene’s F tests and one-way ANOVA’s conducted with post-hoc contrasts (LSD) performed [41].

Stepwise regression was conducted to examine associations between FFE factors and CEBQ scores, adjusting for known covariates in step 1 (parent BMI, child gender, breastfeeding history (binary coded less than 6 months vs more than 6 months), child sleep duration, income (binary coded low-income vs other), region of residency (binary coded Capital City vs other), parent’s depression, anxiety and stress). Coefficients, confidence intervals and mean scores were inspected to check the direction and pattern of the association. All hypotheses will assume a 0.05 significance level and a two-sided alternative hypothesis. All analyses were carried out using SPSS v24 (SPSS Inc., Chicago, IL, USA).

## Results

A sample of 977 participants was obtained from an initial sample of 1186 parents of Australian children, aged between 2.0 and 5.0 years who completed the survey, once cases of biologically implausible values and outliers were removed (as reported in Boswell, et al., 2018) [26]. On comparison with national data, children categorised as underweight appeared to be overrepresented (7.6% vs 22.4%, respectively) [42]. Given the focus of this study on the collective contribution of FFE variables to overweight and obesity in children, in comparison to normal weight children, it was decided to exclude underweight children from further analysis leaving a sample of *n* = 757 [42]. In further support for this approach, a recent systematic review deemed the use of self-reported BMI data as acceptable specifically to screen children for overweight and obesity, with good overall performance with moderate sensitivity and high specificity, but the validity for underweight children is not clear [43]. Excluded cases did not differ significantly based on parent BMI category, parent gender, single parent status, income group, or state or region of residency in one-way ANOVA analysis, however, were significantly younger (mean age 3.1 years, compared with 3.4 years, *p* = 0.000) and a higher proportion were boys (58.0% in excluded case compared with 49.4% in retained sample, *p* = 0.026) (Table 1).

**Table 1:** Demographic Data

	Total sample *n* = 977 (%)	Normal weight, overweigh and obese only *n* = 757 (%)
Child Gender-Boy	483 (49.4)	376 (49.7)
Age		
2 years	108 (11.0)	83 (11.0)
2.5 years	161 (16.5)	128 (16.9)
3 years	153 (15.6)	126 (16.6)
3.5 years	164 (16.8)	120 (15.9)
4 years	173 (17.7)	136 (18)
4.5 years	128 (13.1)	94 (12.4)
5 years	90 (9.2)	70 (9.2)
Child BMI category^a^		
Underweight	219 (22.4)	excluded
Normalweight	586 (59.9)	586 (77.4)
Overweight	109 (11.1)	109 (14.4)
Obese	63 (6.5)	63 (8.2)
Child BMI z-score^b^ (Mean)	− 0.18 (SD 1.79)	0.52 (SD 1.07)
Parent Gender - Male	52 (5.3)	42 (5.5)
Parent BMI category^c^		
Underweight (< 18.50 kg/m^2^)	13 (1.3)	12 (1.6)
Normal weight (18.50–24.99 kg/m^2^)	398 (40.7)	305 (40.3)
Overweight (≥25.00 kg/m2)	254 (26.0)	196 (25.9)
Obese ≥30.00 kg/m^2^)	312 (32.0)	244 (32.2)
Breastfeeding History		
Less than 6 months	358 (36.6)	276 (36.5)
6 months of more	619 (63.4)	481 (63.5)
Income		
Low: less than AU$40,000	129 (13.2)	103 (13.6)
Middle: AU$40,000 - 100,000	407 (41.6)	320 (42.3)
High: more than AU$100,000	441 (45.2)	334 (44.1)
Australian State		
Victoria	173 (17.7)	133 (17.6)
New South Wales	246 (25.2)	189 (25)
Queensland	292 (30.0)	230 (30.4)
Australian Capital Territory	28 (2.9)	21 (2.8)
Western Australian	122 (12.5)	93 (12.3)
Tasmania	29 (3.0)	23 (3.0)
Northern Territory	5 (0.5)	4 (0.5)
South Australia	82 (8.4)	64 (8.5)
Region Type		
Capital city	255 (26.1)	201 (26.6)
Metro (population over 100,000)	301 (30.8)	235 (31.0)
Large rural (population 25,000–99,999)	188 (19.3)	145 (19.2)
Small rural (population 10,000 – 24,999)	128 (13.1)	93 (12.3)
Large remote (population 5000 – 9999)	41 (4.2)	32 (4.2)
Small remote (population less than 5000)	64 (6.5)	51 (6.7)

N (%) reported for dichotomous variables; Mean (SD) reported for continuous

^a^Cut offs per Cole, TJ. (2000 and 2007); ^b^2000 CDC growth charts; ^c^Cut offs per WHO classifications for adults (2000)

Data presented in this table has previously been published in Boswell, et al., (2018)

### Family food environment factors

Eight factors reflecting FFE were extracted from the factor analysis, explaining between 9.37 and 4.89% of the variance in FFE (cumulative variance explained 53.51%). A Kaiser-Mayer-Olkin measure of 0.704 was achieved with significance in Bartlett’s test of sphericity (*p* = 0.000), indicating acceptable correlation (Table 2) [37, 38].

**Table 2:** Varimax- rotated Family Food Environment variables loading on factors extracted^a^

	Component							
	1 Negative Feeding Strategies	2 Negative Nutrition Related Beliefs	3 High Resources	4 High Skill	5 Health focused restriction	6 Family Meals	7 TV and devices	8 Parent’s nutrition Knowledge
Belief: Healthy Eating is expensive		**0.712**						
Belief: It takes too long to prepare healthy food		**0.696**						
Belief: Healthy Food doesn’t taste good		**0.583**						
Belief: Healthy eating is important					**0.650**			
Suitable cooking facilities			**0.878**					
Suitable food storage			**0.870**					
Sufficient money to buy food each week		**−0.562**	**0.397**					
Parent’s shopping skills				**0.754**				
Parent’s cooking skills				**0.836**				
Single Family Meal^b^						**0.683**		
Food as a reward for eating^b^	**0.817**							
Food as a reward for behaviour^b^	**0.698**							
Parent use of Persuasive feeding^b^	**0.717**							**−0.272**
Parent use of Covert restriction^b^					**0.723**			
Parent use of Overt restriction^b^	**0.437**				**0.464**			
Structured Meal setting^b^	**0.359**					**0.421**		
Structured Meal timing^b^					**0.302**	**−0.353**	**−0.332**	
Frequency of Family Meals per week						**0.630**		
Parent Total Nutrition Knowledge								**0.833**
Family use of TV/devices during meals (yes/sometimes)						**−0.303**	**0.362**	**−0.416**
Child use of TV/devices during meals (yes/sometimes)							**0.682**	
Adult use of TV/devices during meals (yes/sometimes)							**0.677**	
Availability of fruit and vegetables (Sometimes/never)		**0.383**		**−0.260**			**0.292**	

Extraction Method: Principal Component Analysis

Rotation Method: Varimax with Kaiser Normalization

a. In interest of table readability, family food environment variables loading < 0.25 and <-0.25 are not shown

b. FPSQ-28 Sub Scales (Jansen, et al. 2016)

### Family Food Environments & Child BMI category

A statistically significant difference existed between child BMI categories (normal weight, overweight and obese) for four FFE factors in the MANOVA, with Pillais’ Trace = 0.046, F(16, 1498) = 2.22, *p* = 0.004. The multivariate effect size was estimated at 0.023, which implies that 2.3% of the variance in the dependent variables was accounted for by BMI categories. Based on a series of Levene’s F tests, the homogeneity of variance assumption was considered satisfied for all factors except Factor 2, however, as of the largest standard deviations were not more than four times the size of the corresponding smallest standard deviation, it was considered, in accordance with Howell, (2007), that ANOVA would be robust [44].

ANOVA’s for Factor 1 ‘Negative Feeding Strategies’, Factor 2 ‘Negative Nutrition Related Beliefs’, Factor 7 ‘Use of TV and Devices’, and Factor 8 ‘Parent’s Nutrition Knowledge’ were statistically significant (*p* = 0.046, *p* = 0.004, *p* = 0.049 and *p* = 0.032, respectively), with effect sizes (partial n^2^) ranging from 0.008 to 0.015 (Table 3). In post-hoc analyses (Fisher’s LSD) examining individual mean difference in factor scores across BMI categories, statistically significant differences were seen in Factor 1 ‘Negative Feeding Strategies’ between normal weight and obese (*p* = 0.017) and overweight and obese (*p* = 0.026), such that obese children scored highest on this factor. Factor 2 ‘Negative Nutrition Related Beliefs’ differed significantly between normal weight and overweight (*p* = 0.003) and between normal weight and obese (*p* = 0.047), such that normal weight scored lower on this factor. Factor 7 ‘Use of TV and Devices’, differed significantly between normal weight and obese (*p* = 0.032) such that normal weight scored higher on this factor. Factor 8 ‘Parent’s Nutrition Knowledge’ differed significantly between normal weight and obese (*p* = 0.012) and between overweight and obese (*p* = 0.017), such that obese scored lower on this factor (Table 3).

**Table 3:** Family Food Environment factor differences by child BMI category (MANOVA)

Family Food Environment Factors*	Normal Weight (*n* = 586) Means (SD)	Overweight (*n* = 109) Means (SD)	Obese (*n* = 62) Means (SD)	Sig. (*p* value)	Partial n2
Factor 1: Negative feeding strategies^1^	−0.021 (1.0)	− 0.056 (0.95)	0.296 (0.98)	**0.046**	0.008
Factor 2: Negative Nutrition Related Beliefs^2^	−0.065 (1.0)	0.239 (0.98)	0.196 (1.13)	**0.004**	0.015
Factor 3: High Resources^3^	−0.009 (1.0)	0.132 (0.87)	−0.136 (1.15)	0.209	0.004
Factor 4: High Skill^4^	0.011 (0.99)	−0.072 (1.05)	0.033 (1.03)	0.658	0.001
Factor 5: Health Focused Restriction^5^	−0.000 (1.02)	−0.048 (0.97)	0.088 (0.87)	0.690	0.001
Factor 6: Family Meals^6^	−0.000 (1.00)	0.027 (0.97)	−0.041 (1.01)	0.901	0.000
Factor 7: TV and Devices^7^	0.045 (1.01)	−0.106 (1.03)	− 0.238 (0.83)	**0.049**	0.008
Factor 8: Parent’s Nutrition Knowledge^8^	0.021 (0.98)	0.065 (1.05)	−0.311 (1.02)	**0.032**	0.009

*Factor characteristics:

1**:** Food as a reward for eating, Food as a reward for behaviour, Parent use of Persuasive feeding, Parent use of Overt restriction, Structured Meal setting,

2: Belief: Healthy Eating is expensive, Belief: It takes too long to prepare healthy food, Belief: Healthy Food doesn’t taste good, Availability of fruit and vegetables, Sufficient money to buy food each week (negatively loaded)

3: Suitable cooking facilities, Suitable food storage, Sufficient money to buy food each week

4: Parent’s shopping skills, Parent’s cooking skills, Availability of fruit and vegetables (negatively loaded)

5: Belief: Healthy eating is important, Parent use of Covert restriction, Parent use of Overt restriction, Structured Meal timing,

6: Single Family Meal, Structured Meal setting, Structured Meal timing (negatively loaded), Frequency of Family Meals per week, Family use of TV/devices during meals (negatively loaded)

7: Structured Meal timing (negatively loaded), Family use of TV/devices during meals, Child use of TV/devices during meals, Adult use of TV/devices during meals, Availability of fruit and vegetables

8: Parent use of Persuasive feeding (negatively loaded), Parent Total Nutrition Knowledge, Family use of TV/devices during meals (negatively loaded)

### Relation between FFE factors and children’s eating behaviours, controlling for covariates

After controlling for covariates in step 1, ‘Negative Feeding Strategies’ was positively associated with food fussiness (β = 0.201, *p* = 0.001), and food responsiveness (β = 0.305, *p* = 0.000), ‘Negative Nutrition Related Beliefs’ was positively associated with food responsiveness (β = 0.117, *p* = 0.018), and ‘Parent’s Nutrition Knowledge’ was negatively associated with slowness in eating (β = − 0.108, *p* = 0.031). No CEBQ sub-scales were significantly associated with ‘TV and devices’ (Table 4).

**Table 4:** Variables Predictive of Family Food Environment Factors, controlling for covariates (excluding UW)

	Factor 1: Negative Feeding Strategies		Factor 2: Negative Nutrition Related Beliefs		Factor 7: TV and devices		Factor 8: Parent’s Nutrition Knowledge	
*Step 1-*Covariates	B (SE)	Β (*P* Value)	B (SE)	Β (*P* Value)	B (SE)	Β (*P* Value)	B (SE)	Β (*P* Value)
Parent BMI	−0.793 (0.321)	− 0.110 (0.014)	1.658 (0.318)	0.226 (0.000)				
Child sex (male)			0.179 (0.087)	0.087 (0.040)				
Breastfeeding less than 6 months			0.182 (0.090)	0.087 (0.044)			−0.237 (0.099)	−0.111 (0.017)
Child Sleep duration								
Low Income			0.400 (0.119)	0.147 (0.001)			−0.380 (0.129)	−0.138 (0.003)
Parent Anxiety								
Parent Depression								
Parent Stress							0.350 (0.141)	0.155 (0.014)
Capital City Residency					0.236 (0.109)	0.099 (0.031)	0.270 (0.112)	0.114 (0.015)
*Step 2-*Children’s Eating Behaviour Questionnaire Sub-scales								
Enjoyment of Food								
Food Fussiness	0.214 (0.065)	0.201 (0.001)						
Food Responsiveness	0.410 (0.068)	0.305 (0.000)	0.160 (0.067)	0.117 (0.018)				
Satiety Responsiveness								
Slowness in Eating	0.237 (0.069)	0.161 (0.001)					−0.164 (0.076)	−0.108 (0.031)

## Discussion

The current study greatly extends on previous research by deriving factors of FFE variables to more authentically examine how a broad scope of interpersonal and microenvironment influences (aligned with the socio-ecological model) combine and relate to the development of obesity and obesogenic eating behaviours during early childhood. Only two previous studies have been identified which similarly attempted to derive factors or clusters of FFE variable to examine the combine effect of variables on obesity development, however, these studies limited their focus to dining times, physical activity/play time, and screen time, which does not capture the range of variables conceptualised within the FFE as described in this paper [45, 46].

This study specifically found four of the eight FFE factors derived to be associated with child BMI category. Scores for Factor 1 ‘Negative Feeding Strategies,’ and Factor 2 ‘Negative Nutrition Related Beliefs,’ were seen to increase across increasing BMI category (normal weight, overweight and obese), while scores for Factor 7 ‘Use of TV and devices’, and Factor 8 ‘Parent’s Nutrition Knowledge’, were seen to decrease. The relationship between these factors with BMI category were in the expected direction (as will be discussed) in all cases except for Factor 7 ‘Use of TV and Devices’, which, in accordance with the literature, could be expected to relate to an increased weight status due to the impact TV use is reported to have on satiety signals and food cue responsivity (through exposure to food advertising) [8, 47]. Additionally, the limited use of structured meal timing, as also loaded on this factor, could be considered a detrimental aspect of children’s nutrition environments that theoretically contributes negatively to a child’s BMI [8, 31, 47]. That is, lack of structure around meal times fails to establish the routine and predictability that underpins responsive feeding practices, as associated across the literature with detrimental eating behaviours and obesity development [48]. This unexpected direction of the relationship between Factor 7 ‘Use of TV and devices’ may in part be explained by the positive association between this factor and residing in a capital city (Table 4), which is generally associated with more positive health outcomes and may be related to a variety of other ‘protective’ factors. Irrespective of this, this relationship requires further investigation, particularly in light of changing uses of technology whereby exposure to food advertising may be less pertinent.

Likewise, while it was expected that higher nutrition knowledge of parents (Factor 8) would be associated favourably with child BMI, as seen in this study, the literature reflecting this appears inconsistent [49–53]. This inconsistency across the literature in part may be attributed to the difficulty in measuring nutrition knowledge, however, it may also be due to nutrition knowledge acting as a proxy for more ‘advantaged’ life circumstances, as supported by the findings of this study which shown Factor 8 ‘Parent’s Nutrition Knowledge’ to be associated with residing in a capital city, breastfeeding for more than 6 months and not identifying as of low income. The effect of such ‘advantaged’ life circumstances may further be reflected in the positive association between ‘Parent’s Nutrition Knowledge’ (Factor 8) and slowness in eating, as a food avoidance eating behaviour associated with a reduced obesity risk, although, in previous analysis of these data slowness in eating was not significantly associated with child BMI [3, 26, 54]. Alternatively, given that use of persuasive feeding strategies, as a ‘non-responsive’ feeding practice associated with childhood obesity, and family use of TV during meals, as discussed to be detrimental, also loaded negatively onto Factor 8, the inconsistencies previously seen in the literature between nutrition knowledge and weight status, could be due to failing to consider other aspects of the FFE that work synergistically with nutrition knowledge and support the translation of knowledge into health behaviours [48].

Given the strength of the relationship between non-responsive feeding practices and childhood obesity, as seen in a systematic review of 31 studies, 20 of which specifically involved children during early childhood, the relationship between Factor 1 ‘Negative Feeding Strategies’ and child BMI category in this study is generally not surprising [48]. What is interesting in relation to Factor 1 ‘Negative Feeding Strategies,’ however, is that the feeding practice *structured meal setting*, as a responsive feeding practice hypothesised to allow children to eat in a setting in which they can attend to their internal cues of hunger and satiety, also loaded onto this factor. This somewhat contradictory finding in this study, again highlights the importance of considering FFE variables as composites, reflecting an authentic environmental exposure. In this instance, while the hypothesise of a structured meal setting allowing a child to attend to their hunger and satiety cues holds much merit, the findings of this study suggest that when combined with other non-responsive feeding practices such as overt restriction and food as a reward, the overall impact on a child’s weight and eating behaviours (namely food fussiness and food responsiveness; Table 4) is negative. This could be due to the overall context and family climate in which such structure around meal settings is imposed, as consistent with the idea of authoritarian parenting (high control, rigidness, low responsiveness) which has similarly been seen to be associated with childhood obesity as well as the use of a range of non-responsive feeding practices [55, 56].

Of further interest, Factor 1 ‘Negative Feeding Strategies’ was seen to be negatively associated with parent BMI, although no other psycho-social variables. This negative association with parent’s BMI, in the absence of other psycho-social variables which would contribute to a high risk factors for childhood obesity, is likely to be particularly important in highlighting the increased risk of obesity development imposed on children by parents who implement non-responsive feeding strategies irrespective of psycho-social risk [57]. Tripicchio, et al., (2014), similarly showed this, by demonstrating that after controlling for shared environment and genetics, restrictive feeding practices were associated with child weight status [58]. The findings of this study are further consistent with the literature which has shown non-responsive feeding strategies to relate to eating behaviours such as food responsiveness and food fussiness [10, 12, 13].

While the intention of parents in implementing such non-responsive feeding practices are likely well intended, given the bi-directional relationship between feeding practices and children’s eating behaviours, particularly food responsiveness as shown by Jansen, et al., (2018), and associated with Factor 1 (Negative Feeding Strategies), it is plausible that parents implement such non-responsive feeding strategies in an attempt modulate eating behaviours and/or control child weight [59]. Additionally, given that Factor 1 was also associated with food fussiness, it is possible that a similar bi-directional relationship food fussiness and strategies intended to overcome such difficult meal time behaviours occurs. Jansen, et al., (2017) and Harris, et al., (2016), have specifically shown the presence of a bi-directional relationship with non-responsive feeding practices and fussy food behaviour in young children [23, 60]. Given this likely misdirection of parent’s good intentions in feeding their child, intervention strategies which focus on providing support for parents to understand and interpret their child’s individual tendencies/innate eating behaviours, as well as implement the appropriate responsive feeding strategies, are likely to be of importance in reducing obesity development and/or in modifying obesogenic eating behaviours.

On this note, in addition to examining the cumulative impact of FFE variables on children’s eating behaviours and obesity development, this study provides insight into the relationship between psycho-social variables and FFE factors which may contribute to understanding of inequitable obesity risk within the population and directly extends on our previous research which explored the relationship between children’s eating behaviour with psycho-social variables, as were hypothesised to impact upon children’s eating behaviours and obesity development through neuro-biological pathways [26]. This extended perspective on the contribution of FFE variables on the development of obesity, and the examination of psycho-social variables associated with these factors, is likely to be of benefit in planning obesity prevention interventions which are more appropriately targeted, in consideration of authentic FFE exposure. For instance, obesity prevention initiatives focusing on constructs aligning with the cumulative use of ‘Negative Feeding Strategies’ may have general suitability to early childhood populations since no demographic variables were associated with this factor. In more specifically targeting lower socio-economic populations, however, children of obese parents, and/or boys in particular, framing interventions towards variables cumulatively associated with the factor ‘Negative nutrition beliefs,’ such as availability of fruits and vegetables within the home and parental skills to prepare quick, healthy, tasty and affordable meals, may be more appropriate. Longitudinal studies are additionally needed, however, to better inform such future directions particularly given that during the early childhood period obesity is still emerging and as such alternative FFE factors could have differing longitudinal impacts on obesity development. Factor 3 ‘High Resources’ and Factor 4 ‘High Skills,’ for instance, may have an obesity protective effect longitudinally that was not seen cross-sectionally in this study.

### Strengths and weaknesses

This study captures a broad scope of variables conceptualised within the FFE and uniquely considers the collective influence of these variables on childhood obesity development. Whilst this study is limited in its exclusion of physical activity measures, the inclusion of children’s eating behaviours is a strength given the significant relationship between CEBQ sub-scales and obesity development, as is the inclusion of parent’s feeding practices and strategies, as a pivotal socio-cultural influence widely examined for its role in the development of obesity and eating behaviours [2, 3, 12, 13, 18, 45, 46, 61, 62]. On this note, the use of the CEBQ, the FPSQ-28, as well as the DASS-21, adds strength to this study as these measures have been well validated across the literature [27, 28, 30, 31]. Caution in interpretation of these results should, however, still be taken due to several survey items being adapted specifically for this study which may compromise validity.

In this regard, the inclusion, and consequently significance of, parent’s nutrition-related beliefs is a unique and important aspect of this study. Little attention has been given in the literature to understanding the role of parent’s specific beliefs and attitudes towards food and nutrition, which, based on the findings of this study, play a significant role in the development of obesity and obesogenic eating behaviours. Whilst the effect of these beliefs on child weight was seen cumulatively and alongside other FFE variables in this study, the specificity of the beliefs measured is highly informative in terms of understanding current facilitators of nutrition-related behaviours as well as opportunities to consequently support behaviour change. Further attention in the literature should be given to exploring this role of parent’s nutrition-related beliefs on child weight and eating behaviours through use of additionally validated measures.

While the inclusion of a broad range of covariates in this study allows for a thorough picture of psycho-social influences on FFEs of children during early childhood in Australia, it is recognised that this may increase the risk of type 1 errors. While no adjustments were made for this, it can be seen in Table 4 that the many of *p*-values are quite low and as such the interpretation of the majority of results would not differ with adjustment. Despite being cross-sectional in nature, this study is strengthened by the large sample of participants representing all states and territories in Australia. Single parents were represented at a rate comparable to the 15% reported nationally, and distribution of participants in the high and middle income groups were represented similarly, although low income families were underrepresented [63]. This under-representation of low income families is likely to be a limitation of this study which impacts the generalisability and application of these results, particularly in obesity prevention initiatives. Although anthropometric data in this study were self-reported, steps were taken to ensure the biological plausibility of included cases, as is considered a quality feature given that approximately 41% of large epidemiological studies do not address biological implausibility [64]. Similar to what has been reported in other studies, anthropometric data deemed biologically implausible values was higher in boys, although, contrary to other studies implausible data were higher in younger children [65, 66]. No differences in demographic characteristics were between children classified as underweight compared with other BMI categories.

On this note, although underweight children were excluded from analysis in this study, the decision to do so is well justified. Firstly, the overrepresentation of underweight children in the initial sample compared to national data (22.4% vs 7.55%, respectively) would likely have further compromised the generalisability of the findings of this study [42]. Furthermore, given the focus of this study on obesity development, the comparability of overweight and obese children to national date (15.2% overweight and 5.5% obese, at 4–5 years of age), once underweight children were removed, strengthens the validity of the results. [63, 67] On this note, the over-representation of underweight children in the original sample could be in part attributed to recruitment through social media which biased the sample. This sample bias may also assist to explain rates of breastfeeding longer than 6 months (63.4%) being higher than national average (50% still receiving breastmilk at 6–9 months) [68]. This risk of sample bias is important to consider in interpreting the results of this study.

## Conclusion

Environmental factors within the FFE have a clear relationship with the development of childhood obesity and obesogenic behaviours. Consideration of the composite effect of FFE on these outcomes is likely to be important in guiding future research and obesity prevention initiatives by providing a more authentic picture of the FFE children are exposed to, from which more targeted and appropriate strategies can be developed. Examining factors of FFE variables in conjunction with psycho-social variables, as in this study, further articulates the reciprocal influence of these variables on environmental constructs thus assisting in understanding of inequitable distribution of obesity risk. Acknowledging the different and multiple needs of sub-populations in this manner may be used to better tailor obesity prevention interventions.

## Abbreviations

BMI: Body Mass Index
CEBQ: Children’s Eating Behaviour Questionnaire
DASS: Depression, Anxiety and Stress Scale
FFF: Family Food Environment
FPSQ: Feeding Practice and Structure Questionnaire
RRMA: Rural, Remote and Metropolitan Areas
TV: Television
